# SET7/9-dependent methylation of ARTD1 at K508 stimulates poly-ADP-ribose formation after oxidative stress

**DOI:** 10.1098/rsob.120173

**Published:** 2013-10

**Authors:** Ingrid Kassner, Anneli Andersson, Monika Fey, Martin Tomas, Elisa Ferrando-May, Michael O. Hottiger

**Affiliations:** 1 Institute of Veterinary Biochemistry and Molecular Biology, University of Zurich, Winterthurerstrasse 190, 8057 Zurich, Switzerland; 2 Life Science Zurich Graduate School, Molecular Life Science Program, University of Zurich, Winterthurerstrasse 190, 8057 Zurich, Switzerland; 3 Department of Biology, Bioimaging Center, University of Konstanz, Universitätstrasse 10, 78464 Konstanz, Germany; 4 Department of Physics, Center for Applied Photonics, University of Konstanz, Universitätstrasse 10, 78464 Konstanz, Germany

**Keywords:** ADP-ribosylation, lysine methylation, PARP-1, protein regulation, SET7/9

## Abstract

ADP-ribosyltransferase diphtheria toxin-like 1 (ARTD1, formerly PARP1) is localized in the nucleus, where it ADP-ribosylates specific target proteins. The post-translational modification (PTM) with a single ADP-ribose unit or with polymeric ADP-ribose (PAR) chains regulates protein function as well as protein–protein interactions and is implicated in many biological processes and diseases. SET7/9 (Setd7, KMT7) is a protein methyltransferase that catalyses lysine monomethylation of histones, but also methylates many non-histone target proteins such as p53 or DNMT1. Here, we identify ARTD1 as a new SET7/9 target protein that is methylated at K508 *in vitro* and *in vivo*. ARTD1 auto-modification inhibits its methylation by SET7/9, while auto-poly-ADP-ribosylation is not impaired by prior methylation of ARTD1. Moreover, ARTD1 methylation by SET7/9 enhances the synthesis of PAR upon oxidative stress *in vivo*. Furthermore, laser irradiation-induced PAR formation and ARTD1 recruitment to sites of DNA damage in a SET7/9-dependent manner. Together, these results reveal a novel mechanism for the regulation of cellular ARTD1 activity by SET7/9 to assure efficient PAR formation upon cellular stress.

## 2. Background

ADP-ribosyltransferase diphtheria toxin-like 1 (ARTD1, formerly named PARP1, [1]) is a nuclear protein that post-translationally modifies proteins by transferring the ADP-ribose moiety from NAD^+^ to specific amino acid residues of target proteins. It is the best described member of the ADP-ribosyltransferase (ART) protein family, which currently comprises 22 human enzymes [1]. ARTD1 is not only the main nuclear ART, but also the primary acceptor for polymeric ADP-ribose (PAR). ARTD1 can be ADP-ribosylated at specific lysine residues and is also modified by acetylation and sumoylation between the amino acid residues 481 and 525 [2–4]. Protein modification with a single ADP-ribose unit or with PAR chains regulates protein function and is implicated in biological processes such as transcriptional control, cell differentiation or cell-cycle regulation [5,6]. Many cellular functions of ARTD1 are brought about by complex formation with partner proteins or the ADP-ribosylation of target proteins in the cell nucleus [5,7]. For example, histones or transcription factors are poly-ADP-ribosylated (PARylated) by ARTD1, which causes concomitant changes in chromatin structure and DNA metabolism [8,9].

Genotoxic and cellular stresses activate ARTD1 enzyme activity [10]. However, the detailed upstream mechanisms leading to the activation of ARTD1 and the involvement of PTMs-modulating ARTD1 activity are little understood. *In vitro*, the DNA-dependent interaction between the amino-terminal DNA-binding domain and the catalytic domain of ARTD1 increased *V*max and decreased the *Km* for NAD^+^ [4]. The amount of DNA in this study was kept at a saturating 1 : 1 ratio (DNA : ARTD1 dimer). It is currently not clear whether ARTD1 activity and the subsequent PAR formation under non-saturating DNA levels depend on additional regulatory mechanisms.

SET7/9 (also called Setd7 or KMT7) was discovered as a histone methyltransferase that causes monomethylation of histone 3 lysine 4 (H3K4me1) [11] and is thereby involved in the regulation of euchromatic gene expression [12–14]. However, SET7/9 has only weak activity on nucleosomes [15], which implies that the main targets of the enzyme are non-histone proteins. In agreement with this hypothesis, numerous non-histone proteins such as Dnmt1 (reduction in stability), p53 (activation and stabilization), TAF10 (increased affinity for polymerase II), oestrogen receptor α (activation and stabilization), pRb, p65, MyoD and Tat protein of HIV1 are methylated by SET7/9 [16–24]. In addition, a recent study identified up to 90 new non-histone SET7/9 target peptides and a strong methylation of free H2A and H2B tails [25]. This promiscuous targeting of different substrates by SET7/9 suggests a low specificity of the enzyme. SET7/9 knockout mice are viable and fertile and loss of SET7/9 does not seem to impair p53-dependent cell-cycle arrest or apoptosis following DNA damage [26,27], although SET7/9 was originally thought to regulate p53 activity in human cells [16]. SET7/9 preferentially modifies positively charged amino acid regions and methylates the last lysine residue in the motif [K>R] [S>KYARTPN] [K] [25]. Peptides that do not perfectly match this sequence can be methylated to a lesser extent. In cells, a strong interaction of acceptor proteins with the SET7/9 methyltransferase might stimulate the transfer of a methyl group to weak target sites. Hence, a weaker methylation does not have to imply a lower biological importance [25].

SET7/9-mediated monomethylation of non-histone proteins is a reversible PTM that can be removed by demethylases such as the lysine-specific demethylase 1 (LSD1) [28,29] and likely also by the close homologue LSD2. Both proteins are flavin-dependent demethylases that are specific for mono- and dimethylated lysines and which are part of histone modification complexes that control cell-specific gene expression [30,31].

The study presented here identifies ARTD1 as a new SET7/9 target protein that is methylated at K508, which enhances PAR synthesis upon oxidative stress. Similarly, SET7/9 also affected PAR synthesis and ARTD1 recruitment to sites of DNA damage *in vivo* upon laser irradiation. These results define methylation of ARTD1 by SET7/9 as an additional regulatory element for cellular ADP-ribosylation and ARTD1 enzymatic activity.

## 3. Results and discussion

### 3.1. ARTD1 is methylated *in vitro* and *in vivo* at K508 by SET7/9

**Figure 1.**
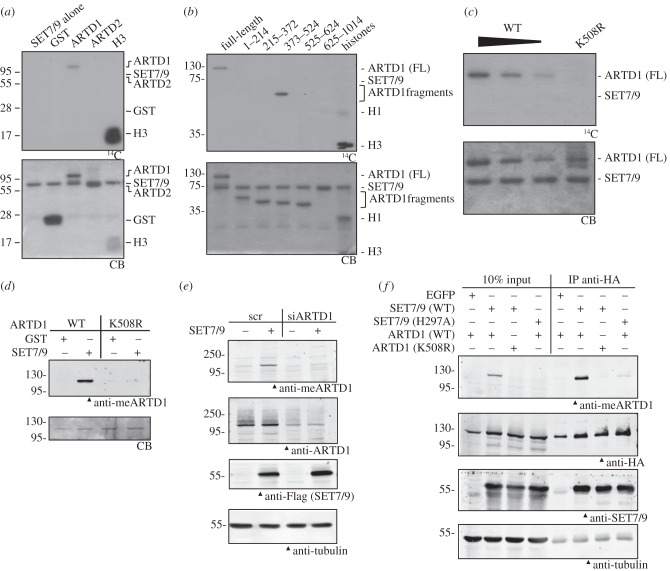
ARTD1 is methylated at K508 by SET7/9 *in vitro* and *in vivo*. (*a*) GST, ARTD1, ARTD2 and H3 were incubated with SET7/9 and ^14^C-labelled SAM in an *in vitro* methylation assay, separated by SDS-PAGE and analysed by autoradiography (^14^C). Coomassie blue (CB) stained gels are shown below. (*b*) Full-length ARTD1 and fragments covering the whole protein were incubated in an *in vitro* methylation assay and analysed by autoradiography. (*c*) Decreasing amounts of WT ARTD1 and K508R ARTD1 were methylated by SET7/9 and analysed by autoradiography. (*d*) An antibody directed against a peptide carrying the methylated lysine residue of ARTD1 was generated and tested in a western blot with *in vitro* methylated ARTD1 WT and K508R. (*e*) U2OS cells were transfected with scrambled siRNA (scr) or siRNA directed against ARTD1. One day later, cells were transfected with an empty vector or with a plasmid containing WT *SET7/9*. Whole cell extracts were analysed by western blot on day 3 after knockdown using the same antibody as in (*d*). (*f*) U2OS cells were co-transfected with HA-ARTD1 (WT or K508R) and EGFP or Flag-HA-SET7/9 (WT or H297A). After immunoprecipitation with an anti-HA antibody, whole cell extracts and IP samples were analysed by western blotting with the indicated antibodies. All experiments were repeated at least twice, gave a similar result, and one representative blot is shown.

Based on methylation profile searches and preliminary experiments, it was hypothesized that SET7/9 directly methylates ARTD1. To determine whether SET7/9 indeed modifies ARTD1, biochemical *in vitro* methylation assays with purified proteins were performed. SET7/9 methylated the known substrate histone H3 as well as full-length ARTD1, while neither GST nor ARTD2, another member of the ARTD family, was modified (figure 1*a*). To localize the modification site, purified ARTD1 fragments covering the whole amino acid sequence were methylated by SET7/9 *in vitro* (figure 1*b*). The potential SET7/9 modification site(s) in ARTD1 could be narrowed down to the auto-modification domain (AD) consisting of amino acids 373–524, which was strongly methylated *in vitro*, while all other tested ARTD1 fragments (containing the DNA-binding (DBD), WGR or catalytic (CAT) domains) were not methylated (figure 1*b*). *In silico* analysis identified lysine 508 (K508) as the putative target site as it was the only lysine residue within this region matching the published [KR] [STA] [K(me)] consensus motif for SET7/9-dependent methylation [18]. Mutation of K508 to arginine (K508R) indeed abolished SET7/9-dependent methylation of full-length ARTD1 (figure 1*c*). ARTD1 K508 was confirmed as the target residue of SET7/9 by mass spectrometric analysis of recombinant ARTD1 (373–524) *in vitro* methylated by SET7/9 (see electronic supplementary material, figure S1*a*,*b*). To confirm methylation of ARTD1 K508 in cells, a polyclonal antibody against a synthetic human ARTD1 peptide containing monomethylated K508 was generated. The anti-meARTD1 antibody specifically recognized the monomethylated peptide (see electronic supplementary material, figure S1*c*) and full-length ARTD1 that was methylated by SET7/9 *in vitro* (see electronic supplementary material, figure S1*d*), while the methylation-deficient K508R mutant was not detected (figure 1*d*). *In vivo*, the same antibody specifically detected the methylation of ARTD1 in cells overexpressing SET7/9 (figure 1*e*,*f*). The antibody did not detect methylation of overexpressed mouse ARTD1 in mouse cells, which was most probably owing to sequence differences between human and mouse ARTD1 at the methylation site.

These results defined ARTD1 as a new target for SET7/9- dependent methylation *in vitro* and *in vivo* and identified K508 as the main target site for SET7/9-dependent methylation of ARTD1.

### 3.2. ARTD1 auto-modification inhibits its methylation by SET7/9

**Figure 2.**
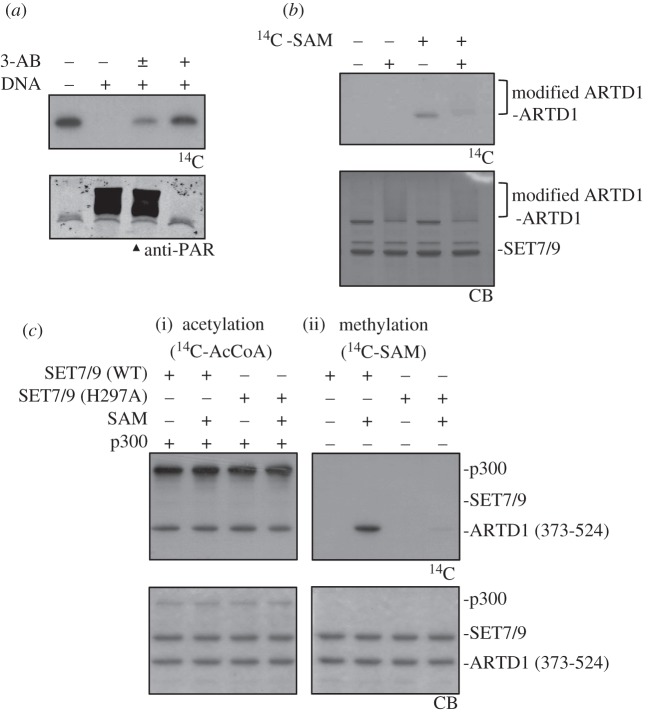
ARTD1 methylation by SET7/9 does not influence its PARylation or acetylation but is inhibited by ARTD1 auto-modification. (*a*) Recombinant ARTD1 was methylated by SET7/9 after ADP-ribosylation in the presence or absence of DNA and 3-AB. ±: 3-AB was added after the ADP-ribosylation reaction. (*b*) ARTD1 was first incubated with SET7/9 in the presence or absence of ^14^C-SAM and afterwards incubated with activating DNA and cold NAD^+^ to allow auto-modification. (*c*) ARTD1 (373–524) was first incubated with WT SET7/9 or an enzymatic dead mutant (H297A) in the presence of cold SAM and afterwards acetylated with p300 and ^14^C-AcCoA (i). Methylation was controlled with ^14^C-SAM (ii). All experiments were repeated at least twice, gave a similar result, and one representative blot is shown.

Interestingly, the SET7/9 target residue K508 lies within a heavily modified region (aa 486–524) of the ARTD1 AD domain that comprises five acetylation and three ADP-ribosylation sites as well as one lysine residue that can be sumoylated (see electronic supplementary material, figure S2). Modification of ARTD1 with SUMO did not affect its ADP-ribosylation activity, but completely abrogated p300-mediated acetylation of ARTD1, revealing an intriguing crosstalk of sumoylation and acetylation on ARTD1 [2]. Crosstalk between different PTMs of the same modified amino acid residue has been documented in particular for modifications comprising the histone code [32–34]. It was thus tested whether there is crosstalk between PARylation, acetylation and SET7/9-dependent methylation of ARTD1 *in vitro*. Prior stimulation of recombinant ARTD1 with DNA in the presence of NAD^+^ and subsequent auto-modification completely inhibited methylation by SET7/9 (figure 2*a*). Inhibition of ADP-ribosylation by 3-aminobenzamide from the beginning (3-AB; +) reverted this effect on ARTD1 methylation, while 3-AB addition after auto-modification, but before addition of SET7/9 (3-AB; ±), still resulted in markedly decreased methylation (figure 2*a*). Consequently, these experiments suggested that auto-ADP-ribosylation of ARTD1, but not a possible ADP-ribosylation of SET7/9 by ARTD1, prevented subsequent methylation. The sharp band of methylated ARTD1 running at the height of unmodified ARTD1 strengthened the conclusion that SET7/9 only methylated ARTD1 that was not or only slightly ADP-ribosylated.

By contrast, auto-PARylation of ARTD1 was not impaired by prior methylation of ARTD1 as indicated by the smear of methylated ARTD1 upon incubation with cold NAD^+^ and DNA (figure 2*b*). Similarly, methylation of the 373–524 ARTD1 fragment by SET7/9 did not affect subsequent acetylation by p300 (figure 2*c*(i)). The experiment was controlled with the enzymatically inactive H297A SET7/9 mutant and methylation of ARTD1 was confirmed in a parallel experiment using ^14^C-SAM (figure 2*c*(ii)).

These results suggested that SET7/9-dependent methylation of ARTD1 is influenced by ARTD1 auto-modification, while neither PARylation itself nor acetylation by p300 is impaired by the methyl-modification of K508.

### 3.3. SET7/9-dependent methylation stimulates ARTD1 activity

**Figure 3.**
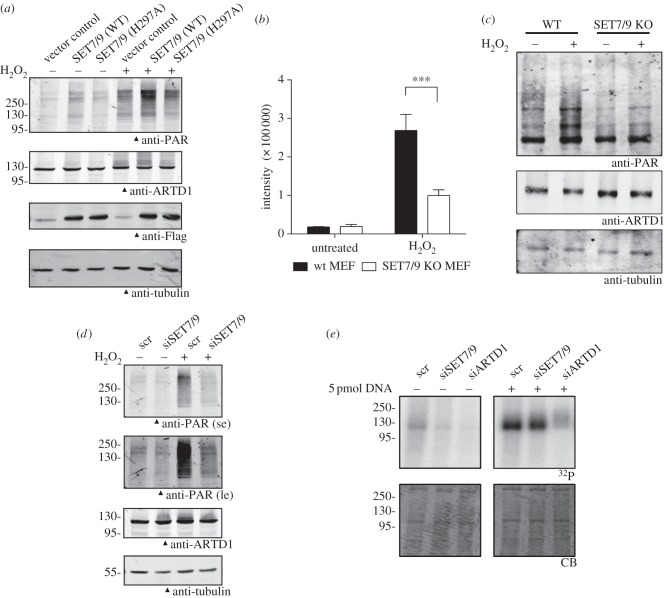
SET7/9-dependent methylation increases ARTD1 activity in cells. (*a*) After overexpression of Flag-SET7/9 WT or H297A, U2OS cells were treated with or without 1 mM H_2_O_2_ for 5 min and PAR formation was analysed by western blot. (*b*) H_2_O_2_-induced PAR formation was analysed by immunofluorescence in SET7/9 KO and WT MEFs. The intensity of the anti-PAR-stained cells was quantified. (*c*) H_2_O_2_-induced PAR formation in SET7/9 KO and WT MEFs was analysed as in (*a*). (*d*) U2OS cells were transfected with scrambled siRNA (scr) or siRNA targeting SET7/9. Three days after knockdown, H_2_O_2_-induced PAR formation was analysed as in (*a*). Short and long exposures (se and le, respectively) of the anti-PAR blot are shown. (*e*) ARTD1 activity was analysed in NEs from U2OS cells after knockdown of SET7/9 and ARTD1 for 3 days by radioactive ADP-ribosylation assays. All experiments were repeated at least twice, gave a similar result, and one representative blot is shown. Quantifications are shown in the electronic supplementary material, figures S3*b* and S4*c*,*d*.

In order to test the hypothesis that SET7/9 regulates the enzymatic activity of ARTD1 *in vivo*, we first confirmed that both enzymes are localized in the nucleus of U2OS cells. While ARTD1 was only present in the nucleus and enriched in the nucleoli, SET7/9 was localized throughout the cell except in the nucleoli (see electronic supplementary material, figure S3*a*). Next, Flag-tagged wild-type (WT) SET7/9 was overexpressed and PAR formation following oxidative stress by H_2_O_2_ was determined (see figure 3*a* and electronic supplementary material, figure S3*b*). PAR formation was indeed increased upon overexpression of WT SET7/9 (lanes 2 and 5), even in unstimulated cells (lane 2), while the enzymatically inactive SET7/9 mutant H297A did not cause this effect (lanes 3 and 6). To prove that SET7/9 stimulated PAR formation, we analysed mouse fibroblasts lacking SET7/9. Upon H_2_O_2_ stimulation, SET7/9-knockout MEFs showed significantly reduced PAR staining and lower PAR-synthesizing activity as compared with the WT control cells (see figure 3*b*,*c* and electronic supplementary material, figure S3*c*), suggesting that SET7/9 regulates PAR formation *in vivo*. This was also confirmed by SET7/9 knockdown in U2OS cells (see electronic supplementary material, figure S4*a*,*b*). Following oxidative stress by H_2_O_2_, siSET7/9-treated cells formed less PAR than cells transfected with a control siRNA (see figure 3*d* and electronic supplementary material, figure S4*c*). To further analyse the influence of SET7/9 on ARTD1 enzymatic activity in cells, nuclear extracts (NEs) from siRNA-treated U2OS cells (control siRNA or siRNA directed against SET7/9 or ARTD1) were prepared and auto-ADP-ribosylation of ARTD1 was tested *in vitro* in the presence or absence of exogenous DNA. Downregulation of SET7/9 reduced the basal ARTD1 activity to levels only slightly above those in siARTD1 cells (in the absence of exogenous DNA, figure 3*e*; electronic supplementary material, figure S4*d*). This effect was also seen, but to a lesser extent, when ARTD1 activity was stimulated by an excess of exogenous DNA, suggesting that SET7/9 methylation regulates ARTD1, especially in the absence of a strong stimulus.

These results suggested that SET7/9-dependent methylation stimulates ARTD1-dependent PAR formation in U2OS cells.

### 3.4. SET7/9-dependent methylation of ARTD1 at K508 regulates ADP-ribosylation *in vivo*

**Figure 4.**
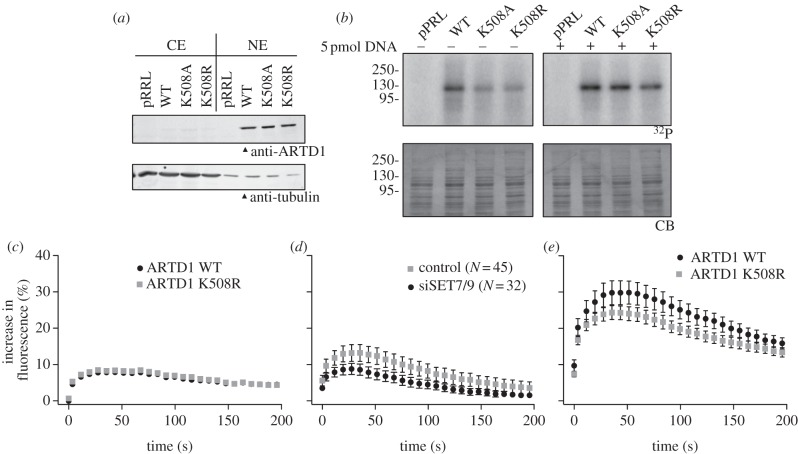
Methylation-deficient ARTD1 is less active and less efficiently recruited to sites of local DNA damage induced by femtosecond laser irradiation. (*a*) ARTD1 knockout MLFs were stably complemented with WT ARTD1 or two methylation-deficient mutants. Cells were then fractionated and CEs and NEs were analysed by western blot. (*b*) ARTD1 activity in NE from (*a*) was analysed by radioactive PAR assays in the absence or presence of 5 pmol-activating DNA. Experiments in A and B were repeated twice with a similar outcome and one representative blot is shown. Quantifications are shown in the electronic supplementary material, figure S5*a*,*b*. (*c*) Recruitment of WT and K508R ARTD1 to sites of local DNA damage induced by femtosecond laser irradiation at *λ* = 1050 nm. (*d*) Recruitment of macroH2A1.1-EGFP to sites of local DNA damage induced by femtosecond laser irradiation at *λ* = 1050 nm. (*e*) Recruitment of WT and K508R ARTD1 to sites of DNA damage by femtosecond laser irradiation at *λ* = 775 nm.

To elucidate whether SET7/9-dependent methylation of ARTD1 at K508 is directly responsible for the observed influence of SET7/9 on ARTD1-dependent PAR formation *in vivo*, *ARTD1−*/− MLFs were stably genetically complemented with WT ARTD1 or with two methylation-deficient mutants (K508A and K508R). The WT and the mutant proteins were comparably expressed in the NEs, but not detectable in the cytoplasmic extracts (CEs) or the vector control (pRRL) (figure 4*a*). NEs containing WT or mutant ARTD1 were incubated with radioactively labelled NAD^+^, but without exogenous DNA, and ARTD1 auto-ADP-ribosylation was assessed. The methylation-deficient ARTD1 mutants K508A and K508R exhibited markedly reduced activity in comparison with the WT control (see figure 4*b* and electronic supplementary material, figure S5*a*). Upon addition of excess DNA, the methylation-deficient ARTD1 proteins K508A and K508R still exhibited reduced enzymatic activity, but the effect was less pronounced as compared with conditions without exogenous DNA (see figure 4*b* and electronic supplementary material, figure S5*b*), again pointing at a SET7/9 methylation effect on ARTD1 activation.

This indicated that the methylation-deficient ARTD1 mutants (K508A and K508R) are enzymatically less active and provided further evidence that SET7/9-dependent methylation of ARTD1 at K508 affects its activity.

### 3.5. Mutation of K508 affects ARTD1 recruitment to damaged chromatin

The results described above suggested that SET7/9-dependent methylation of ARTD1 at K508 regulates its enzymatic activity at basal conditions of low levels of DNA damage and in response to oxidative stress (figures 1*c* and 4*b*). We hypothesized that SET7/9-dependent methylation may influence ARTD1 activity by affecting its interaction with chromatin, but SET7/9 downregulation had no effect on the extraction of ARTD1 under different salt concentrations (see electronic supplementary material, figure S5*c*), suggesting that the affinity of ARTD1 to undamaged chromatin was not changed. However, methylation by SET7/9 may prime ARTD1 for efficient recruitment to sites of DNA damage. In order to study whether ARTD1 methylation affects its recruitment to sites of DNA damage *in vivo*, cells expressing EGFP-tagged WT and methylation-deficient ARTD1 were analysed by localized femtosecond laser irradiation [35]. This method allows studying the kinetics of the recruitment of proteins to sites of DNA damage. The nature of the lesion can be influenced via the irradiation wavelength: at 775 nm, both DNA strand breaks and UV photoproducts are generated, while at 1050 nm mainly DNA strand breaks are produced [35]. Irradiation with a wavelength of 1050 nm caused similar recruitment of WT and K508R ARTD1 (figure 4*c*) and the release of WT and K508R ARTD1 from the irradiated sites was comparable (data not shown), suggesting that methylation did not affect recruitment to sites of DNA damage. However, laser microirradiation of live cells [36] caused significantly lower recruitment of GFP-labelled macroH2A1.1 in siSET7/9 cells as compared with WT (figure 4*d*), which is indicative of reduced PAR synthesis or different ADP-ribose structures. In contrast to irradiation with 1050 nm, femtosecond pulses at *λ* = 775 nm caused lower recruitment of the K508R ARTD1 mutant (figure 4*e*). It is thus possible that SET7/9 differentially affects ARTD1 stimulation depending on the stress level and type of induced DNA damage. In summary, these findings show that SET7/9-dependent methylation stimulates ARTD1 enzymatic activity in response to both oxidative and DNA stress.

## 4. Conclusion

The results presented here suggest that SET7/9 methylates ARTD1 *in vivo* and *in vitro* at lysine K508. The residue K508 was identified as the target site for SET7/9-dependent methylation by site-directed mutagenesis and mass spectrometry, as well as with a specific polyclonal antibody raised against this methylated site. Methylation of ARTD1 by SET7/9 did not prevent its consecutive ADP-ribosylation, but affected ARTD1 recruitment to sites of local DNA damage *in vivo*. Prior auto-ADP-ribosylation of ARTD1 impaired its methylation by SET7/9. Knockdown of SET7/9 or the expression of methylation-deficient ARTD1 in cells lacking WT ARTD1 caused reduced PAR formation *in vitro* and *in vivo*. Moreover, overexpression of SET7/9, but not of its enzymatically inactive mutant enhanced PAR formation in untreated (basal) and H_2_O_2_-treated cells. These findings identify ARTD1 as a new SET7/9 methylation target and reveal a previously unknown mechanism for the regulation of ARTD1 activity in cells.

The stimulatory effect of ARTD1 methylation on PAR formation was most apparent if no exogenous DNA was added to the reactions that were performed with NEs of SET7/9 knockdown cells or of cells genetically complemented with a methylation-deficient ARTD1 mutant (the NEs may contain low amounts of endogenous DNA). The effect was much weaker under conditions of saturating DNA concentrations (DNA : ARTD1 dimer ratio greater than 1). Methylation by SET7/9 may thus represent a priming step that precedes and facilitates the activation of ARTD1 by DNA or comprises a DNA damage independent ARTD1 co-regulatory mechanism. We have already provided evidence that DNA double strand breaks are recognized and bound by the DBD of ARTD1, which subsequently leads to binding to the CAT domain, induces structural changes within the catalytic cleft, increases the affinity for NAD^+^ and stabilizes reaction intermediates [4]. The identified SET7/9-dependent methylation site at K508 of ARTD1 lies within the central AD [4]. It is at the first glance surprising that ARTD1 is methylated in the AD and not in one of the zinc fingers of the DNA-binding domain or in the catalytic domain of ARTD1, but nevertheless affected in its enzymatic activity. However, the AD harbours the ADP-ribose acceptor sites indicating that this domain has to enter the catalytic cleft of ARTD1 to be subsequently modified. SET7/9-dependent methylation may sensitize ARTD1 for auto-modification by stabilizing the AD in the catalytic domain of ARTD1 under non-genotoxic conditions or in the presence of minimal DNA damage (fewer DNA lesions compared with ARTD1 molecules). Alternatively, methylation might induce structural changes, which affect the binding of the DBD to the CAT, and thus sensitize the enzyme for a special type of DNA damage (see below). Likewise, methylated ARTD1 could exhibit higher affinity for its substrate NAD^+^, and therefore show increased catalytic activity. Based on this hypothesis, methylation of ARTD1 at K508 by SET7/9 serves as a sensitization step that assures basal ARTD1 stimulation to assure PAR formation upon oxidative stress. The fact that we observed a similar effect of SET7/9 on the irradiation-induced PAR formation does not necessarily imply a similar regulatory mechanism. However, in order to compare such regulatory mechanisms and to study ARTD1 stimulation by SET7/9 mechanistically, structural analyses would be required.

The AD represents a PTM hotspot that is also modified by ADP-ribosylation, acetylation and sumoylation [2–4]. Interestingly, prior auto-modification of recombinant ARTD1 inhibited subsequent methylation by SET7/9 most probably through steric hindrance (figure 2*a*), which is in agreement with earlier studies providing evidence that the adjacent lysines 498, 521 and 524 are the acceptor sides for ADP-ribose [4]. Similarly, a synthetic ARTD1 peptide acetylated at K508 could not be methylated by SET7/9 (see electronic supplementary material, figure S5*d*). However, the SET7/9-mediated methylation of ARTD1 did not inhibit its auto-ADP-ribosylation, indicating that the methylation would not interfere with the positioning of this domain into the catalytic domain. The functional relevance of this crosstalk needs to be further defined. It is intriguing to speculate that ARTD1 auto-modification would hamper K508 methylation to avoid an additional enhancement of its activity through this modification. Moreover, this could explain the inefficiency of SET7/9-dependent methylation to further activate already stimulated ARTD1 and hints again at a sensitization function of SET7/9 for ARTD1 under non-stimulatory conditions.

The presence of SET7/9 had no influence on the overall affinity of ARTD1 for (undamaged) chromatin *in vivo*. By contrast, different recruitment of WT and K508R ARTD1 to sites of local damage in the nucleus was observed. Interestingly, WT and K508R ARTD1 showed similar recruitment to DNA lesions induced with a wavelength of 1050 nm, while a clear reduction was observed for the mutant after treatment with laser pulses at 775 nm (figure 4*c*,*e*). This effect was likely owing to the UV photoproducts generated at 775 nm or to other differences in the types of lesion induced by 775 nm versus 1050 nm irradiation. The latter wavelength mainly induces DNA strand breaks but achieves a lower overall level of damage than 775 nm at the irradiation conditions used here [35]. Alternatively, a higher affinity for a certain type of lesion or chromatin alteration (qualitative difference) of the methylated ARTD1 protein, as compared with the unmethylated or the non-methylatable mutant, could also contribute to this behaviour.

The methylation of ARTD1 *in vivo* is very difficult to detect. This indicates either low endogenous levels of ARTD1 K508 methylation or further di- and trimethylation at this residue by other methyl transferases. Here, SET7/9 strongly affected ARTD1 activity in the presence of low amounts of DNA and upon stimulation by H_2_O_2_, although we do not know whether this was due to oxidative damage of the DNA. ARTD1 and its enzymatic activity are also important for chromatin compaction [7,37]. An increased ARTD1 activity might lead to a more open chromatin, allowing subsequent histone modifications (epigenetic events) changing the chromatin status and structure. Furthermore, our studies provide evidence for the involvement of SET7/9 in the oxidative stress response of the cell. Whether SET7/9 is similarly required for the response to other signals (e.g. N-methyl-N′-nitro-N-nitrosoguanidine or phorbol 12-myristate 13-acetate) remains to be investigated. The fact that SET7/9 is not required for cell-cycle arrest or p53 stabilization in mice suggests that the methylation-dependent stimulation of PAR formation is not required for these aspects but serves for other, yet to be identified, signalling pathways. Most importantly, the results presented here may indicate DNA-damage independent induction of ARTD1 activity *in vivo* and suggest that ARTD1 methylation stimulates ADP-ribosylation in response to other cellular stresses that do not necessarily involve DNA damage [5].

## 5. Material and methods

### 5.1. Plasmids and protein expression

pGEX-SET7/9 (52–366) and pcDNA3-SET7/9 (full-length/WT and H297A) were kindly provided by D. Reinberg (Howard Hughes Medical Institute, NYU School of Medicine, New York, NY, USA). pcDNA4-Flag-HA-SET7/9 was created by subcloning SET7/9 into pcDNA4. pCMV-HA-PARP1 and pRRL-vectors as described previously [2]. All point mutations were inserted by site-directed mutagenesis. The construct encoding macroH2A-EGFP was kindly provided by A. Ladurner (Department of Physiological Chemistry, Ludwig-Maximilians Universität (LMU) Munich, Munich, Germany).

The baculovirus expression vector BacPak8 (Clontech, Mountain View, CA, USA) was used for the expression of recombinant proteins in Sf21 insect cells, as described previously [38]. Recombinant GST-tagged proteins were expressed in *Escherichia coli*. All recombinant proteins were purified by a one-step affinity chromatography using ProBond resin (Invitrogen, Zug, Switzerland) for His-tagged and glutathione sepharose (GE Healthcare, Zurich, Switzerland) for GST-tagged proteins, according to the manufacturer's recommendations.

### 5.2. Antibodies and siRNAs

The following antibodies were used for immunoblotting: rabbit PARP-1 (H-250, Santa Cruz, Heidelberg, Germany); rabbit poly(ADP-ribose) (LP96–10, BD Biosciences, Allschwil, Switzerland); rabbit SET7/9 (no. 2815, Cell Signalling); mouse Flag (M2, Sigma-Aldrich, Buchs, Switzerland); mouse tubulin (T6199, Sigma); rabbit PARP (mono methyl K508) (ab92986) was generated in collaboration with Abcam (Cambridge, UK) using a synthetic ARTD1 peptide containing the methylated lysine residue (LSKKSK(me1)GQVKE).

The following FlexiTube siRNAs (QIAGEN, Hombrechtikon, Switzerland) were used in RNAi experiments: AllStars Negative Control, Hs_SET7_3 and Hs_PARP1_6.

### 5.3. Tissue culture and transfections

U2OS cells were cultured in Dulbecco's modified eagle medium (PAA Laboratories, Pasching, Austria) supplemented with 10% FCS and penicillin/streptomycin. MLFs were cultured in the same medium supplemented in addition with non-essential amino acids (Gibco/Invitrogen). The SET7/9 knockout MEFs were obtained from Colby Zaph and were previously described [26]. Transfections with the indicated plasmids were performed with TransIT-LT1 (Mirus Bio, Madison, WI, USA) according to the manufacturer's instructions and cells were harvested after 48 h. Knockdown was achieved by reverse transfection of 16 nM siRNA using RNAiMAX (Invitrogen) according to the manufacturer's protocol. Cells were harvested after 3 days. Cells were treated with 1 mM H_2_O_2_ in PBS containing 1 mM MgCl_2_ for 10 min and with 0.5 µM Adr in normal medium.

Complementation of ARTD1 knockout MLFs was achieved by retroviral transduction with pRRL-myc-PARP1 vectors containing a blasticidine resistance marker or the corresponding empty vector. Generation of viruses and transduction of cells were done as described earlier [39].

### 5.4. *In vitro* methylation assays

A 1 µg substrate protein was incubated with 1 µg bacterially purified GST-SET7/9 in the presence of 0.03 µCi [^14^C]-SAM (PerkinElmer) or 0.8 mM cold SAM (Sigma-Aldrich) in methylation buffer (50 mM Tris–HCl pH8.0, 50 mM NaCl, 10% glycerol, 1 mM PMSF and 1 mM DTT) or PAR buffer (see section below) for 1 h at 30°C. Reactions were stopped by boiling in 10× SDS-loading buffer and separated by SDS-PAGE. Gels were stained with Coomassie blue, incubated in 1 M sodium salicylate for 20 min, dried and exposed on X-ray films at −80°C. For mass spectrometric analysis, ARTD1 fragment 373–524 was methylated as described above, separated by SDS-PAGE, excised from the gel and digested with Glu-C. Peptides were analysed by MALDI-MS.

### 5.5. Sequential *in vitro* modification assays

Sequential ADP-ribosylation and methylation assays were performed in PAR buffer (50 mM Tris–HCl, 4 mM MgCl_2_, 250 µM DTT, 1 mg ml^−1^ pepstatin, 1 mg ml^−1^ bestatin and 1 mg ml^−1^ leupeptin). A 10 pmol recombinant ARTD1 was methylated with 1 µg recombinant GST-SET7/9 as described above. The ADP-ribosylation was then started by addition of 5 pmol-activating DNA and 400 µM cold NAD^+^ (Sigma, after methylation with [^14^C]-SAM) or 100 µM NAD^+^ spiked with [^32^P]-NAD^+^ (Perkin Elmer, after methylation with cold SAM). ADP-ribosylation reactions were incubated for 5 min at 30°C, stopped by addition of 10× SDS-loading buffer and proteins were separated by SDS-PAGE. Hot methylation/ADP-ribosylation was assayed by autoradiography of the Coomassie stained, dried gels, whereas cold modifications were controlled by immunoblotting with the indicated antibodies.

In the reverse experiment, 10 pmol ARTD1 was first incubated with activating DNA and 100 µM NAD^+^ for 5 min on ice. 3-AB (Sigma-Aldrich) was added in a concentration of 8 mM to stop the ADP-ribosylation before the methylation was started by addition of 1 µg SET7/9 and [^14^C]-SAM. The activating DNA used in all assays was an annealed double-stranded oligomer (5′-GGAATTCC-3′). For sequential methylation/acetylation, 1 µg ARTD1 fragment (373–524) was methylated as described above. Acetylation was then started by addition of 20 µl HAT reaction mix (50 mM Tris–HCl pH 8.0, 50 mM NaCl, 10% glycerol, 1 mM DTT, 1 mM sodium butyrate, 1 mM PMSF, 0.5 µg p300 and 75 µM [^14^C]-AcCoA) and allowed to proceed for 1 h at 30°C.

### 5.6. Cellular extracts and ARTD1 activity assays

Whole cell extracts were prepared in lysis buffer (50 mM Tris–HCl pH7.5, 400 mM NaCl, 1%Triton and 25 mM NaF), and chromatin fractions were prepared as described elsewhere [40].

NEs from U2OS cells and complemented MLFs were generated as described earlier [41,42]. Five microgram NEs were incubated in 30 µl reaction buffer (50 mM Tris–HCl, 4 mM MgCl_2_, 1 mg ml^−1^ pepstatin, 1 mg ml^−1^ bestatin, 1 mg ml^−1^ leupeptin and 250 nM [^32^P]-NAD^+^ (0.1–0.2 μCi)) in the absence or presence of 5 pmol-activating DNA for 20 min at 30°C. Proteins were separated by SDS-PAGE, and ADP-ribosylation was analysed by autoradiography. Quantifications were done using the software ImageQuant. Alternatively, ADP-ribosylation assays were performed with cold NAD^+^ and modification was assessed by western blotting with anti-PAR antibody.

### 5.7. Induction of local DNA damage and imaging set-up

Local DNA damage was induced by femtosecond laser irradiation, and recruitment of fluorescently tagged proteins was recorded as described previously using an LSM 5 Pascal confocal microscope [35,43]. Briefly, cells were irradiated with femtosecond laser pulses through a 40× oil immersion lens with a numerical aperture of 1.3 (EC-Plan-Neo-Fluar, Carl Zeiss) along a 6 μm track within the nucleus, followed by fluorescence imaging at 488 nm. The maximum peak irradiance in the focal plane was 330 GW cm^−2^ (pulse duration 200 fs, repetition rate 40 MHz) for excitation at 775 nm and 1200 GW cm^−2^ (pulse duration 85 fs, repetition rate 107 MHz) at 1050 nm. Time series of fluorescence images were quantified with ImageJ (http://rsb.info.nih.gov/ij) as described [43,44].

## Supplementary Material

Supplementary Figures
