# Supplementary material for: SET7/9-dependent methylation of ARTD1 at K508 stimulates poly-ADP-ribose formation after oxidative stress
Source: Open Biol. 2013 Oct;3(10):120173. doi: 10.1098/rsob.120173 (PMC3814718; doi:10.1098/rsob.120173)
Supplement: Supplementary Figures [file rsob120173supp1.pdf]

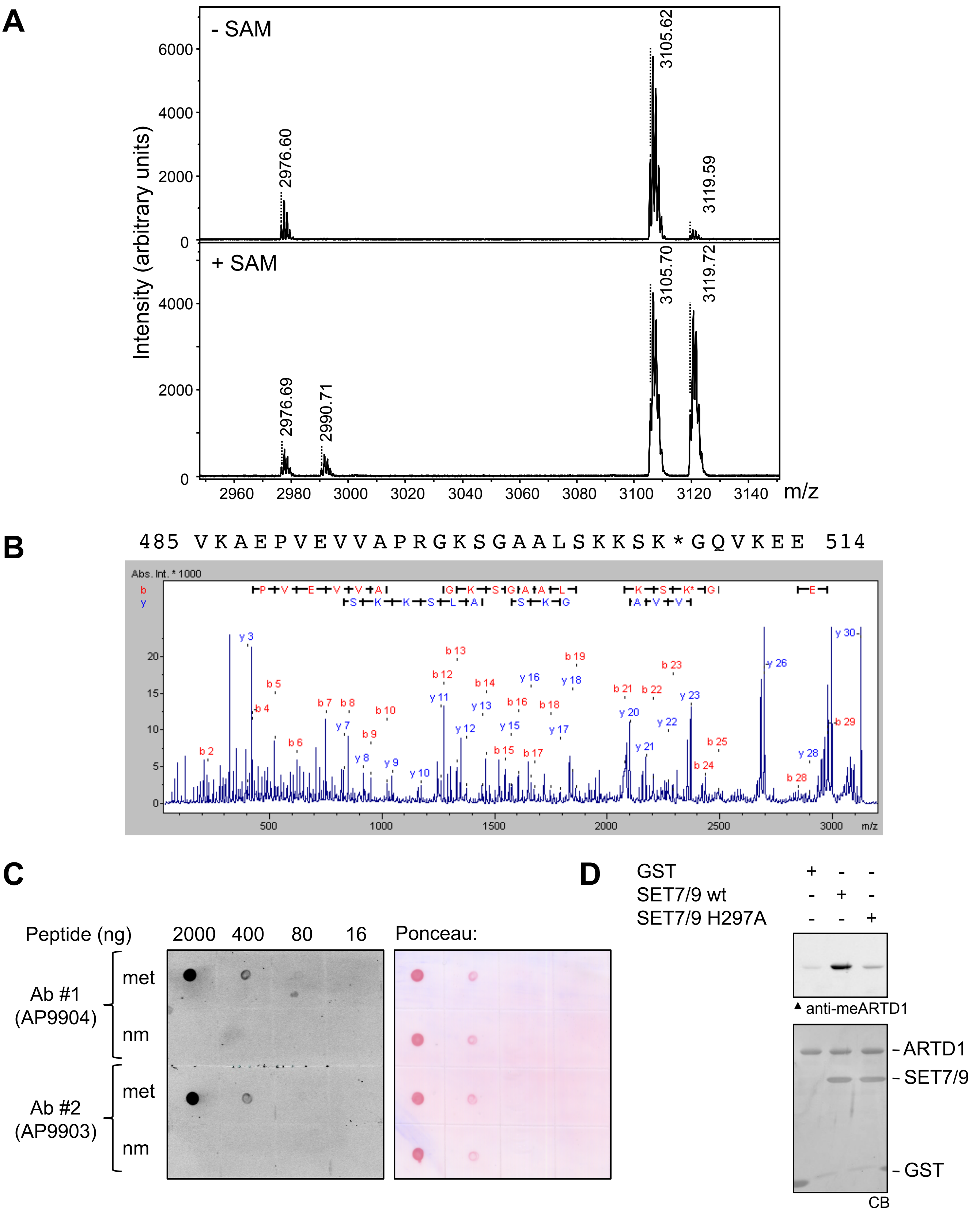

Supplementary Figure 1. (A) Mass spectrometric analysis of recombinant ARTD1 (373-524) incubated with SET7/9 in presence and absence of SAM. (B) MS/MS scan of ion with m/z 3119.72 according to ARTD1 peptide 485 – 514 identifying lysine 508 as the methylated residue. (C) Dot blot with ARTD1 peptides (503-513) not methylated (nm) and methylated (met) at K508 at the indicated concentrations. Two different antibody batches were tested. (D) Validation of a peptide specific antibody directed against monomethylated K508 in ARTD1. A specific signal for K508 ARTD1 methylation was observed upon *in vitro* methylation with WT SET7/9.

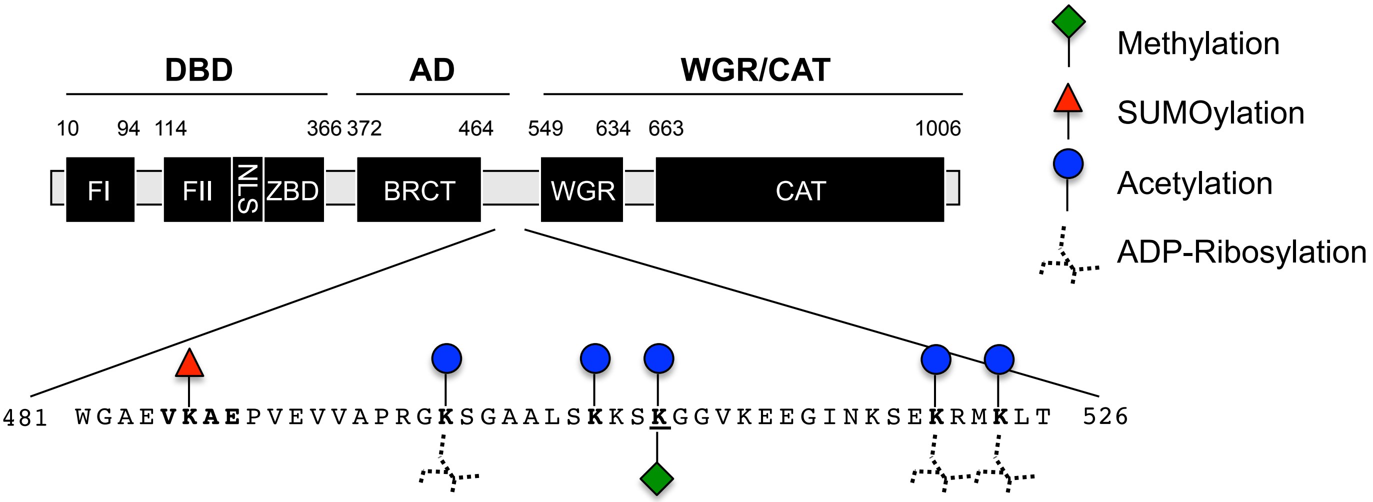

Supplementary Figure 2. Schematic representation of the ARTD1 domain structure and the important methylation, sumoylation, acetylation and ADP-ribosylation sites in the automodification domain of ARTD1.

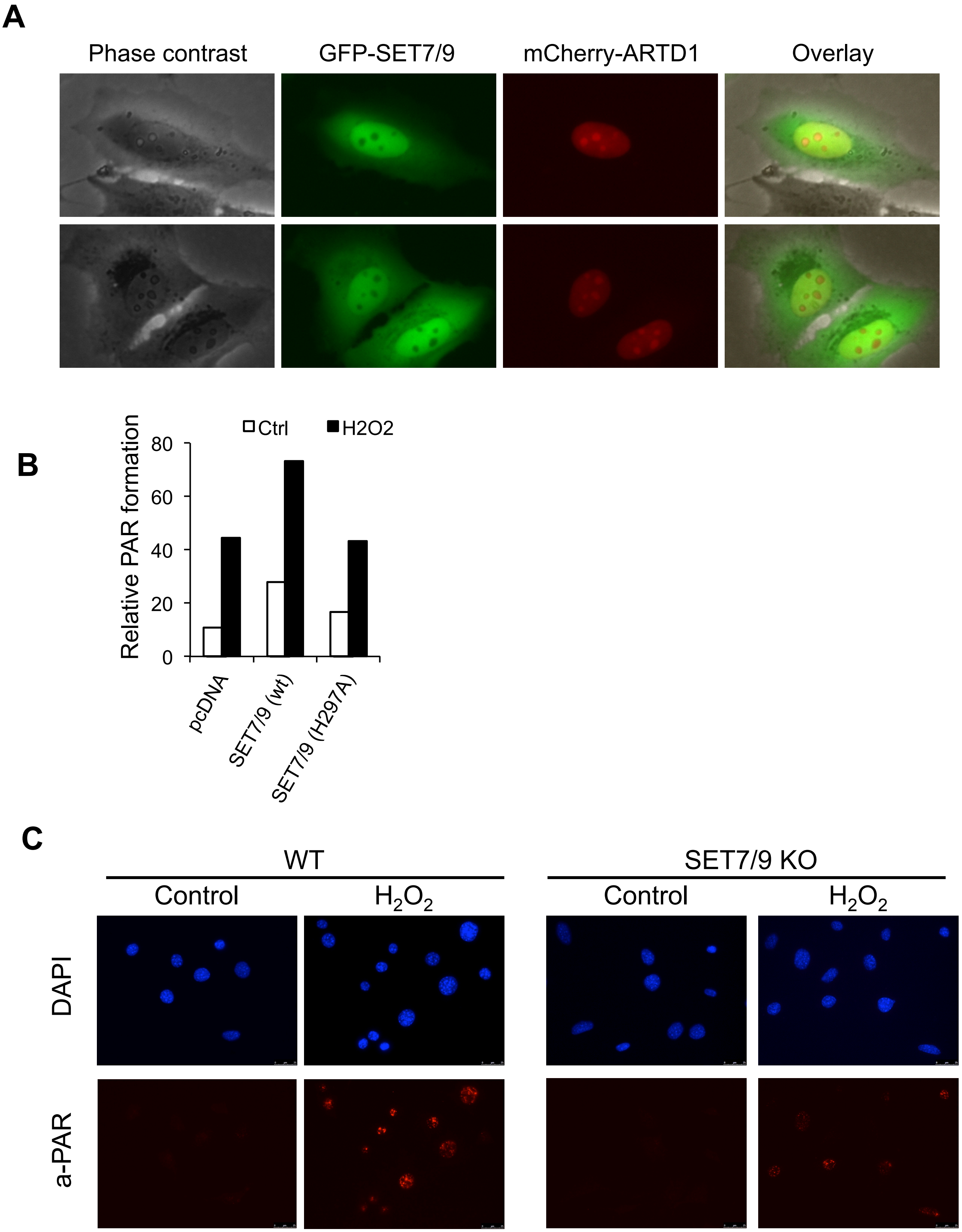

Supplementary Figure 3. (A) U2OS cells expressing GFP-tagged SET7/9 and mCherry-tagged ARTD1 were analyzed by fluorescence microscopy. (B) H_2O_2-induced PAR formation was quantified after overexpression of Flag-SET7/9 WT or H297A. The corresponding western blot is shown in Fig. 3A. (C) WT and SET7/9 KO MEFs were treated for 10 min with 1 mM H_2O_2 and PAR formation was analyzed by immunofluorescence.

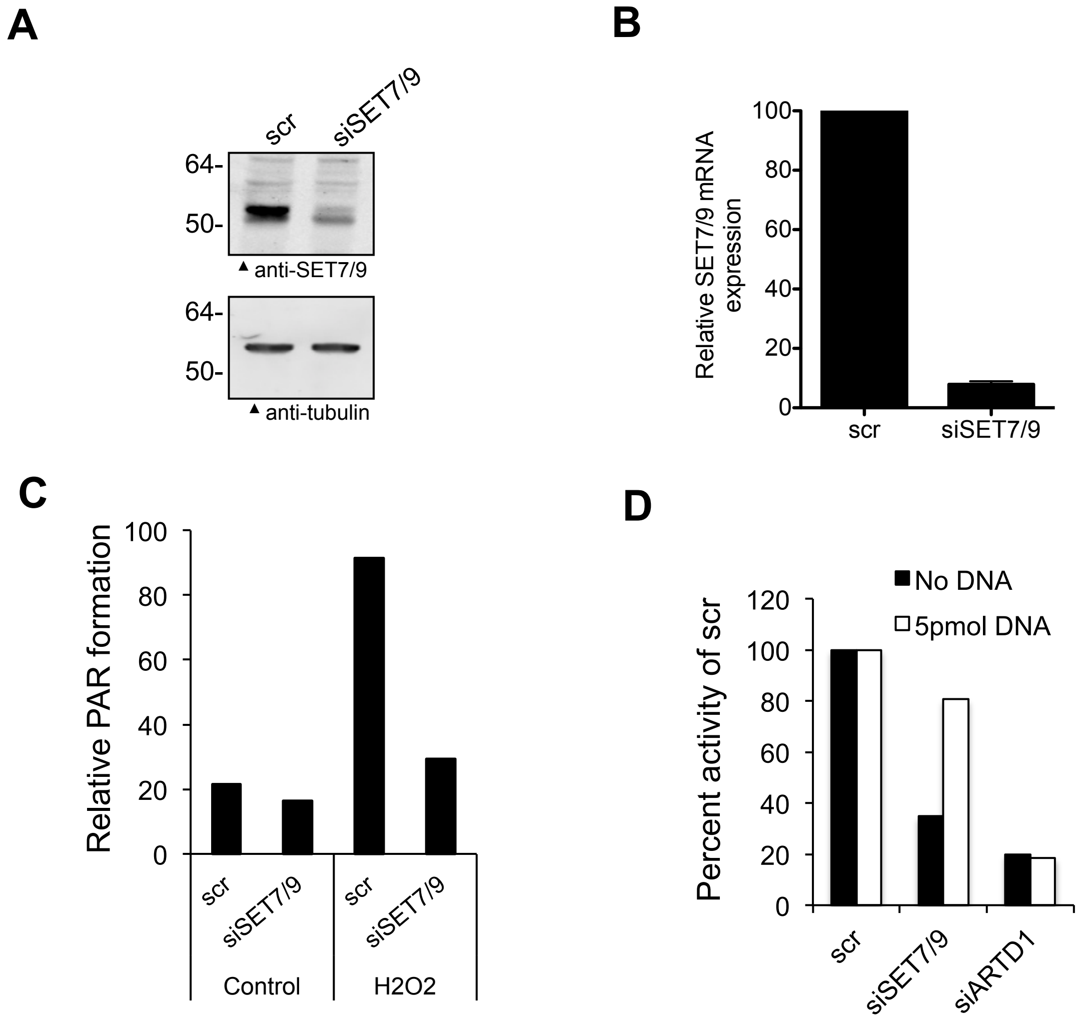

Supplementary Figure 4. (A) U2OS cells were transfected with scrambled siRNA (scr) or siRNA targeting SET7/9. (B) Effect of SET7/9 depletion by siRNA treatment on SET7/9 mRNA levels. The mean of three quantifications and the standard error of the mean are shown. (C-D) U2OS cells were transfected with scrambled siRNA (scr) or siRNA targeting SET7/9. Quantifications of blots shown in Figs. 3D-E. (C) Three days after knockdown, the cells were treated with or without 1 mM H_2O_2 for 5 min and PAR formation was quantified. (D) ARTD1 activity was quantified in nuclear extracts from U2OS cells after knockdown of SET7/9 and ARTD1 for three days.

A

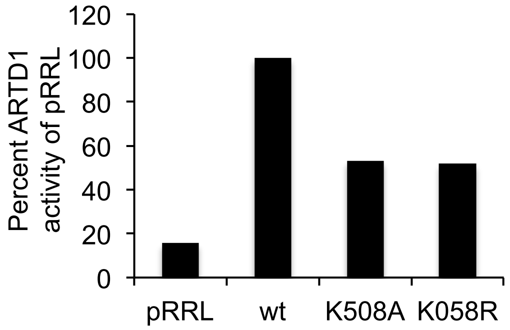

B

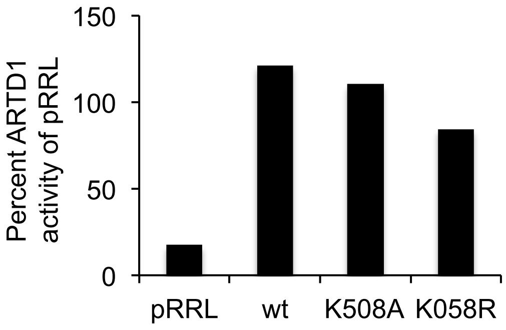

C

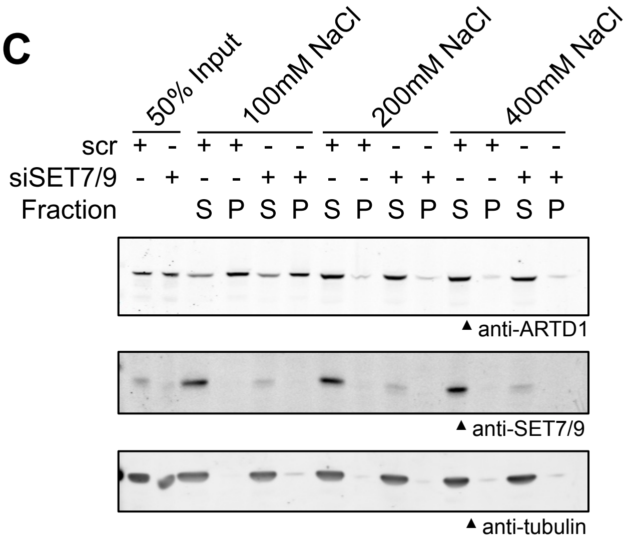

D

Detected modification	Exp. mass	-Ac -SET7/9	-Ac +SET7/9	+Ac -SET7/9	+Ac +SET7/9
None	1548	+	+	-	-
Mono-met	1562	-	+	-	-
Acetyl x3	1674	-	-	+	+
Ac + Met	1688	-	-	-	-

Supplementary Figure 5: (A-B) ARTD1 knockout MLFs were stably complemented with WT ARTD1 or two methylation deficient mutants. Cells were then fractionated and cytoplasmic (CE) and nuclear extracts (NE) were analyzed. Quantifications of western blots shown in Fig. 4B. (A) ARTD1 activity in NE was analyzed by radioactive PAR assay. (B) ARTD1 activity in NE from complemented MLFs as in (A) but in presence of 5 pmol activating DNA. (C) After SET7/9 depletion for three days, ARTD1 affinity to chromatin was tested at different sodium chloride (NaCl) concentrations and analyzed by Western blot. S: supernatant, P: pellet = chromatin bound. (D) Methylation experiment with an ARTD1 peptide and consecutive MS analysis. A synthetic ARTD1 peptide was non-acetylated or acetylated and in vitro methylated with SET7/9. Modifications were detected by MS. Only the non-acetylated peptide was monomethylated in the presence of SET7/9.
